# Blood Cholesterol and Outcome of Patients with Cancer under Regular Cardiological Surveillance

**DOI:** 10.3390/curroncol28010085

**Published:** 2021-02-12

**Authors:** Anna Lena Hohneck, Stephanie Rosenkaimer, Ralf-Dieter Hofheinz, Ibrahim Akin, Martin Borggrefe, Stefan Gerhards

**Affiliations:** 1 University Medical Centre Mannheim, First Department of Medicine (Cardiology), Medical Faculty Mannheim, Heidelberg University, European Centre for AngioScience (ECAS), 68167 Mannheim, Germany; stephanie-luise.rosenkaimer@umm.de (S.R.); ibrahim.akin@umm.de (I.A.); martin.borggrefe@umm.de (M.B.); stefan.gerhards@umm.de (S.G.); 2 DZHK (German Centre for Cardiovascular Research) Partner Site Heidelberg/Mannheim, 68167 Mannheim, Germany; 3 Third Department of Medicine (Oncology), Day Treatment Center (TTZ), Interdisciplinary Tumor Center Mannheim (ITM), Medical Faculty Mannheim, University of Heidelberg, 68167 Mannheim, Germany; ralf.hofheinz@umm.de

**Keywords:** cholesterol, cancer related mortality, cardio-oncology

## Abstract

Cardiovascular (CV) diseases and cancer share several similarities, including common risk factors. In the present investigation we assessed the relationship between cholesterol levels and mortality in a cardiooncological collective. In total, 551 patients receiving anticancer treatment were followed over a median of 41 (95% CI 40, 43) months and underwent regular cardiological surveillance. A total of 140 patients (25.4%) died during this period. Concomitant cardiac diseases were more common in patients who deceased (53 (37.9%) vs. 67 (16.3%), *p* < 0.0001), as well as prior stroke. There were no differences in the distribution of classical CV risk factors, such as hypertension, diabetes or nicotine consumption. While total cholesterol (mg/dL) was significantly lower in patients who deceased (157 ± 59 vs. 188 ± 53, *p* < 0.0001), both HDL and LDL cholesterol were not differing. In addition, cholesterol levels varied between different tumour entities; lowest levels were found in patients with tumours of the hepatopancreaticobiliary system (median 121 mg/dL), while patients with melanoma, cerebral tumours and breast cancer had rather high cholesterol levels (median > 190 mg/dL). Cholesterol levels were significantly lower in patients who died of cancer; lowest cholesterol levels were observed in patients who died of tumours with higher mitotic rate (mesenchymal tumours, cerebral tumours, breast cancer). Cox regression analysis revealed a significant mortality risk for patients with stem cell transplantation (HR 4.31) and metastasised tumour stages (HR 3.31), while cardiac risk factors were also associated with a worse outcome (known cardiac disease HR 1.58, prior stroke/TIA HR 1.73, total cholesterol HR 1.70), with the best discriminative performance found for total cholesterol (*p* = 0.002).

## 1. Introduction

Cardiovascular (CV) diseases and cancer are the leading causes of death worldwide. At first sight, these two entities do not seem to be related to each other, but they share several similarities, including common risk factors (such as smoking, obesity, diabetes mellitus), suggesting a similar underlying pathophysiology [1]. Due to demographic changes leading to an ageing society, the incidence of cancer and CV comorbidities is increasing, which can complicate the choice of the right treatment strategy. This, among other aspects, resulted in the formation of the interdisciplinary field of cardio-oncology [2,3]. Many medical centres are nowadays providing cardiological care for patients undergoing cancer therapy, on the one hand to detect adverse cardiac side effects at an early stage and to modify possible existing risk factors on the other [4]. Therefore, we analysed the impact of common risk factors on mortality, including cholesterol levels, in cancer patients undergoing treatment under regular cardiological surveillance.

## 2. Materials and Methods

In total, 551 patients of the MARCO registry (MAnnheim Registry for CardioOncology) were included in the study. MARCO is a cardio-oncological cooperation which has been established in 2016 at the First Medical Department (Cardiology), University Medical Centre Mannheim, Germany. Written informed consent was obtained from all patients. Patients were followed prospectively.

The registry comprises the collection of demographical and clinical data acquired by a questionnaire and the clinical charts of electronic hospital records, which are updated periodically. 

Both detailed oncological and cardiological history were obtained from each patient. Comorbidities were recorded based on medical history, such as hypertension, hyperlipidemia, diabetes mellitus, and nicotine consumption. Cancer therapies such as anthracyclines, taxanes, monoclonal antibodies or radiotherapy were recorded. 

The study design was conducted according to the principles of the declaration of Helsinki and was approved by the local ethical committee, Medical Ethics Commission II, Faculty of Medicine Mannheim, University of Heidelberg, Germany. Data were analysed anonymously. Data protection was in accordance with the EU Data Protection Directive.

### 2.1. Laboratory Parameters

Total cholesterol (mg/dL), low density lipoprotein (LDL) cholesterol (mg/dL) and high density lipoprotein (HDL) cholesterol (mg/dL) were collected under fasting conditions to provide reliable values.

### 2.2. Endpoints

Mortality from any cause served as primary endpoint during follow-up.

### 2.3. Follow-Up

Patients were followed over a median of 41 (95% CI 40, 43) months. The start date for follow-up was the time of first cancer diagnosis. Follow-up was performed either during routine visits in our outpatient clinic or by telephone contact. Follow-up was available in 97.3% (536 patients).

### 2.4. Statistical Analysis

All data are presented as a mean ± standard deviation, median [interquartile range (IQR)] or numbers (frequencies). Comparisons between two groups were performed using a two-tailed Student’s t-test for parametric and Mann–Whitney U test for nonparametric variables. Categorical variables were compared with the χ2 test. Univariable analysis was performed with linear regression analysis whereby death during follow-up served as dependent variable. Spearman’s correlation coefficient R^2^ was calculated as a coefficient of determination of the correlation. All results were considered statistically significant when *p* < 0.05. Receiver operating characteristic (ROC) curves were used in the whole group of patients to find the optimal cut-off value for total cholesterol, maximising the sum of sensitivity and specificity with help of the Youden-Index.

The influence of different parameters on survival time was investigated using cox regression analysis using block entry of the following variables: age at first diagnosis, metastasised tumour stage, known cardiac disease, prior stroke/TIA, total cholesterol (<152 mg/dL), treatment with 5-FU, radiation, and stem cell transplantation, provided to have a *p* < 0.01 in univariable analysis.

Analyses were performed with Statistical 1 Package for Social Sciences (SPSS for windows 24.0, Chicago, IL, USA) and GraphPad Prism 8.0 (Graphpad Software, Inc., San Diego, CA, USA).

## 3. Results

The study group comprised 551 patients, with a balanced male to female ratio (273 male, 278 female). Mean age at the time of first cancer diagnosis was 61.6 ± 15.1 years. One third (175 patients) had an advanced tumour stage with 48% receiving palliative treatment. Among CV risk factors, arterial hypertension was most frequent (59%), followed by hyperlipidaemia (31%) and smoking history (28%). Diabetes mellitus (17%) and prior stroke or transitory ischemic attack (TIA) (8%) were less common. Approximately 20–30% received concomitant treatment with antihypertensive drugs, anticoagulants or antiplatelets and statins.

During follow-up 140 patients (25.4%) died. Patients who died tended to be older at the time of first diagnosis (64.3 ± 14.1 vs. 60.6 ± 15.3, *p* = 0.01). Advanced tumour stages were associated with a worse outcome (86 (61.4%) vs. 89 (21.7%), *p* < 0.0001). Concomitant cardiac diseases were more common in patients who died (53 (37.9%) vs. 67 (16.3%), *p* < 0.0001), as well as history of stroke or TIA, while there were no differences in hypertension, diabetes or nicotine consumption. Hyperlipidaemia was found more often in patients who were alive during follow-up (31 (22.1%) vs. 138 (33.6%), *p* = 0.01). While total cholesterol (mg/dL) was significantly lower in patients who deceased (157 ± 59 vs. 188 ± 53, *p* < 0.0001), there were no differences in HDL and LDL cholesterol levels. Except of anticoagulants, which were more often prescribed in patients who died (55 (39.3%) vs. 117 (28.5%), *p* = 0.02), concomitant medication was comparable. Patients who died were treated more often with gemcitabine or 5-FU, as well as radiotherapy, as part of palliative treatment. Surgical treatment was performed more often in patients who were alive during follow-up (66 (47.1%) vs. 238 (57.9%), *p* = 0.03). Complete results are shown in Table 1.

**Figure 1:**
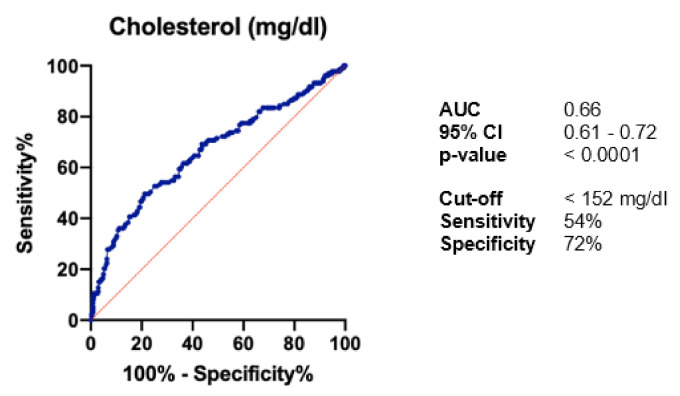
ROC analysis of cholesterol in the whole study population. AUC: area under the curve; CI: confidence interval.

Given the significantly lower cholesterol levels in patients who died, a ROC analysis was performed to find the optimal cut-off value for total cholesterol (with maximising the sum of sensitivity and specificity), corresponding most with an increased mortality risk. A value <152 mg/dL was hereby identified as threshold (sensitivity 54%, specificity 72%; AUC 0.66, *p* < 0.0001) (Figure 1).

**Figure 2:**
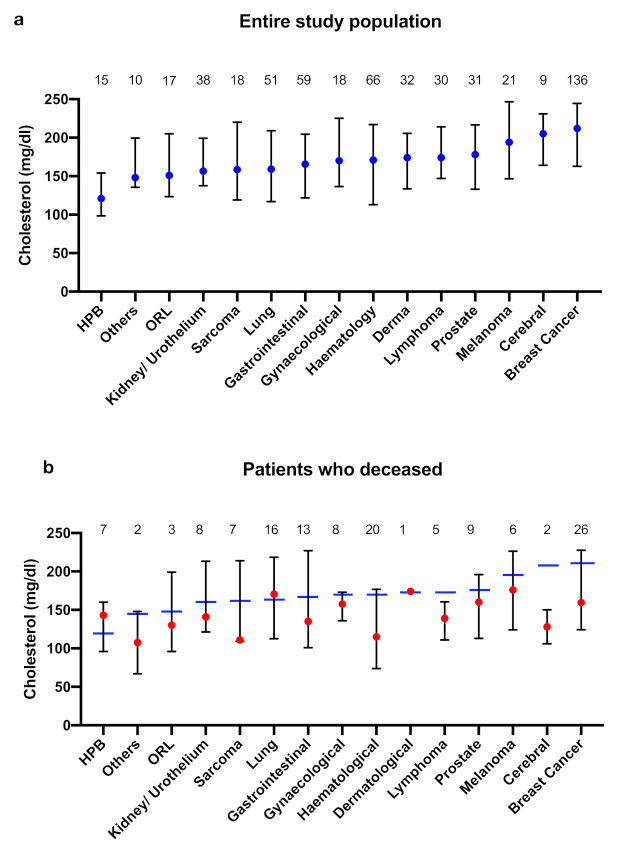
Cholesterol levels based on different tumour entities (**a**) for the entire study population (**b**) for patients who died. Values are presented as median (marked with blue dot or blue bar for the entire study population and marked with red dot for patients who died) and IQR. Absolute number of patients per group are displayed above the bars. HPB: hepatopancreaticobiliary; ORL: otorinolaryngological.

Cholesterol levels were then compared in the overall study population based on different tumour entities, which revealed differences (Figure 2a).

Cholesterol levels of patients with tumours of the hepatopancreaticobiliary system (HPB), otolarnygological (ORL) tumours or rarer tumours (such as germ cell tumours, cancer of unknown primary (CUP); here referred to as “Others”) were below the calculated cut-off of 152 mg/dL. Cholesterol levels of patients with other tumours (gastrointestinal/ gynaecological tumours, lung cancer, etc.) were relatively similar, with values between 160 and 180 mg/dL. However, patients with melanoma, cerebral tumours or breast cancer showed significantly increased cholesterol levels of over 190 mg/dL.

In addition, we compared cholesterol levels of patients who died during follow-up and those who were alive, also based on different tumour entities. In most cases, lower cholesterol levels were observed in patients who died. This was particularly evident, reaching statistical significance, in patients with haematological tumours (*p* = 0.005), lymphoma (*p* = 0.02), breast cancer (*p* = 0.0004), and cerebral tumours (*p* = 0.002) (Table 2). However, cholesterol levels in patients who died of HPB tumours or lung cancer were higher when compared to patients who were alive during follow-up, though not reaching statistical significance.

Lowest cholesterol values (<152 mg/dL) were found in patients who died of mesenchymal tumours (sarcoma, haematological diseases), gastrointestinal, urogenital and HPB tumours. In addition, (female) patients with gynaecological tumours and (male) patients with prostate cancer showed cholesterol levels of about 160 mg/dL, which is comparable to the overall study population. Highest cholesterol levels (>170 mg/dL) were observed in patients with lung cancer and malignant melanoma. Cholesterol levels of patients who deceased are graphically displayed in Figure 2b.

In order to compare the influence of different parameters on survival time, a cox regression analysis, including age at first diagnosis, metastasised tumour stage, known cardiac disease, prior stroke/TIA, total cholesterol (<152 mg/dL, cut-off determined by ROC analysis), treatment with 5-FU, radiation, and stem cell transplantation, was performed. Patients with stem cell transplantation and advanced (metastasised) tumour stage died significantly more frequently (metastasised tumour stage HR 3.31, stem cell transplantation HR 4.31), while neither radiation (HR 0.95) nor age at first diagnosis (HR 1.02) had an influence on survival time. Treatment with 5-FU, as often used in palliative setting failed to reach statistical significance (*p* = 0.26). Cardiac risk factors were all associated with a worse outcome (known cardiac disease HR 1.58, prior stroke/TIA HR 1.73, total cholesterol HR 1.70), with the best discriminative performance found for total cholesterol (*p* = 0.002). Results of the cox regression analysis are outlined in Table 3.

## 4. Discussion

The present study analysed the impact of common risk factors on mortality, including cholesterol, in cancer patients undergoing treatment under regular cardiological surveillance. We hereby revealed the following:

- Cholesterol levels were significantly lower in patients who died.
- Cholesterol levels varied between different tumour entities; lowest levels were found in patients with HPB tumours (median 121 mg/dL), while patients with melanoma, cerebral tumours, and breast cancer had rather high cholesterol levels (median > 190 mg/dL).
- In patients who died lower cholesterol levels were observed in patients with tumours with higher mitotic rate (mesenchymal tumours, cerebral tumours, breast cancer). This was particularly remarkable in patients with breast cancer, although cholesterol levels of the total population of patients with breast cancer were significantly elevated.
- Patients with stem cell transplantation (HR 4.31) and metastasised tumour stages (HR 3.31) showed the highest mortality risk, while cardiac risk factors were also associated with a worse outcome, whereby the best discriminative performance was found for total cholesterol (*p* = 0.002).

In the 1970s, early attempts were made to investigate a potential association between serum cholesterol levels and cancer mortality [5,6,7]. In most of these studies, lower cholesterol levels were found in patients with cancer, but without evidence of an etiological link. We also could confirm significantly lower cholesterol levels in patients who died of cancer. In contrast to these studies, which primarily investigated male individuals, our registry reports on a representative patient cohort, including both male and female patients, as well as different tumour entities, regardless of histological type or stage. This, however, makes it more difficult to detect causal relationships.

Due to the risk of formation of atherosclerotic plaques [8], the role of cholesterol in cardiology has been well investigated. In patients with cancer, however, its role is not elucidated completely. Cholesterol is not only a precursor for several hormones and bile acids, but also plays an essential role in the architecture of the cellular membrane [9]. Therefore, lower cholesterol in patients with cancer levels might reflect a more active stage of the disease with increased utilisation of cholesterol for formation of new cancer cells. In our cohort, low cholesterol levels were found in patients with mesenchymal tumours, cerebral tumours, and breast cancer, all of which have a high mitotic rate that could support this assumption.

Since tumours use cholesterol for cell division, there have been considerations targeting cholesterol and lipoprotein pathways as a new strategy of anticancer treatment [10,11]. The use of statins, which inhibit the HMG-CoA reductase, the key enzyme in cholesterol synthesis, has become indispensable in cardiology for the prevention of CV events [12]. Experimental studies now put in evidence that the use of statins might also have an impact on oncogenic events, such as cell division, tumour growth, and metastatic potential [13,14]. Statins not only modulate the intracellular synthesis of cholesterol; they also alter the cholesterol content of tumour cell membranes [15]. In addition, the oxidation of cholesterol causes an inflammatory response, which is why statins have also an anti-inflammatory effect [16]. 

The described antitumour effects are not exclusively attributed to the administration of statins, but seem to result directly from the cholesterol-lowering effect. In a mouse model, treatment with ezetimibe, which is a NPC1L1 blocker in the gut, that is responsible for dietary cholesterol adsorption, was also associated with inhibition of tumour angiogenesis [17]. In total, 25% of our study population received statins due to hyperlipidaemia. However, there were no differences in the use of statins between patients who died and those who were alive. 

It now seems almost paradoxical that low serum cholesterol levels were associated with increased cancer mortality in our study population, while iatrogenic cholesterol reduction is thought to have antioncogenic effects. One possible explanation for this could be the method of measurement. The determination of the serum cholesterol only reflects the exchange of cholesterol between different cells, which is a complex homeostasis [18]. Therefore, measuring the intracellular cholesterol level and composition might be more important, which can already be determined for scientific purposes, but is not carried out in clinical routine, neither did we perform the measurement in this reported cohort. Presumably, differences of intracellular cholesterol composition between tumour patients and patients receiving a statin as preventive measure could be apparent. This could be part of further clinical investigations. At this stage we can only speculate that patients with statin therapy would have higher intracellular LDL cholesterol levels. To what extent this might affect cell division or even have protective effects in tumour therapy cannot be predicted with certainty.

Regarding cholesterol levels in different tumour entities, we found striking differences. While lowest cholesterol levels were found in patients with HPB, ORL or rarer tumours (germ cell tumours, CUP), patients with melanoma, cerebral tumours, or breast cancer showed significantly increased cholesterol levels of over 190 mg/dL. The prognosis of patients with HPB, ORL as well as rarer tumours such as CUP is poor and these tumours are often associated with impaired food intake, leading to tumour cachexia and undernutrition. Undernutrition seems to occur less frequently in patients with melanoma, cerebral tumours, and breast cancer, which may explain the differences. In addition, cancer therapies can also lead to an increase in cholesterol. Especially oestrogen treatment which is often used in patients with breast cancer leads to increased cholesterol levels [19]. Moreover, cholesterol is not only a major player in lipid metabolism, but also a precursor of steroid hormones and therefore intimately associated with the aetiology of breast cancer [20,21]. This could explain the contradiction why elevated cholesterol levels were found especially in the total population of patients with breast cancer. As steroid hormones are known to regulate cell proliferation in breast cancer, increased cholesterol levels are most likely to reflect increased metabolism of steroid hormones [18]. In contrast to this, significantly lower cholesterol levels were observed in patients with breast cancer who died. Additionally, lower cholesterol levels were found in patients who died, when compared to patients who were alive during follow-up, with haematological diseases, sarcoma, and cerebral tumours. Especially mesenchymal tumours and cerebral tumours have high mitotic rates, as well as aggressive forms of breast cancer. In most cases, cholesterol levels of patients who died were lower than in patients who were alive and in most tumour entities less than 152 mg/dL, the cut-off which has been determined by ROC analysis. However, patients who died of a HPB tumour or lung cancer showed increased cholesterol values in comparison to patients who were alive. These two tumour entities are often associated with cachexia, which similar to anorexia, can lead to hypercholesterolaemia [22]. Unfortunately, we did not perform repeated measurements of cholesterol in all patients. Our observations suggest a parabolic shape, with normal values at the onset of the cancer, lower values at a more active stage and again increasing values in case of tumour cachexia. To confirm these assumptions, prospective long-term observations are needed. Highest cholesterol levels (>170 mg/dL) were found in patients who died of lung cancer or melanoma. These both tumour entities are associated with a poor prognosis and show also an increased mitotic rate. In these entities BRAF inhibitors, CTLA-4 as well as immune checkpoint inhibitors are often part of the therapy strategies. To our knowledge, hypercholesterolaemia has not been described as a side effect. If these substances could be responsible for the increase in cholesterol needs to be clarified in prospective controlled trials. 

Considering similarities for both CV diseases and cancer, we further studied the impact of common risk factors on mortality. Even though, as mentioned before, we could not prove a direct proportional relationship between cholesterol and mortality risk in patients with cancer, total cholesterol (<152 mg/dL) was associated with a significant risk increase (HR 1.70). Concomitant cardiac diseases and prior stroke or TIA were also associated with overall mortality, which makes it difficult to verify a causal link due to competing risks. 

## 5. Conclusions

Cholesterol has emerged as an indispensable surrogate marker in cardiology. While in cardiology the motto is “the lower the better”, low cholesterol in tumour patients seems to reflect strong cell proliferation and thus a more active stage of the disease, which is associated with a worse prognosis. Although cholesterol levels were lower on average in patients who died, further analyses revealed a rather parabolic shape, with normal values at the onset of the cancer, lower values at a more active stage and again increasing values in case of tumour cachexia. The fact that cholesterol is involved in many biochemical processes of both healthy and malignant cells, makes it difficult to use it as a prognostic marker in a cardio-oncological setting. A direct comparison of intracellular cholesterol concentration and composition could provide information about the different mechanisms in both cancer and CV diseases. 

## 6. Limitations

In this analysis we examined only cardiological markers for their prognostic implication, without including oncological prognostic factors. Repeated measurements of cholesterol were not performed in all patients, which is a major limitation of the study. The registry nature of the study can introduce significant selection bias.

## Figures and Tables

**Table 1:** Baseline characteristics, risk factors and medical treatment.

	All Patients (*n* = 551)	Deceased during Follow-Up (*n* = 140, 25.4%)	Alive during Follow-Up (*n* = 411, 74.6%)	*p*-Value (Univariable)	R^2^
Sex, m (%)	273 (49.5)	63 (45.0)	210 (51.1)	0.23	-
Age at first diagnosis	61.6 ± 15.1	64.3 ± 14.1	60.6 ± 15.3	**0.01**	0.01
Median follow-up (months, 95% CI)	41 (40, 43)	23 (18, 29)	43 (42, 45)	**<0.0001**	0.06
Advanced tumour disease (metastasised)	175 (31.8)	86 (61.4)	89 (21.7)	**<0.0001**	0.14
Relapse	44 (8.0)	19 (13.6)	25 (6.1)	0.9	-
Secondary tumour	91 (16.5)	27 (19.3)	64 (15.6)	0.31	-
Palliative treatment	263 (47.7)	112 (80.0)	151 (36.7)	**<0.0001**	0.14
Karnofsky-Index	77.3 ± 18.9	74.1 ± 19.8	78.4 ± 18.5	**0.02**	0.01
**Shared risk factors**, ***n* (%)**					
Hypertension	324 (58.8)	83 (59.3)	241 (58.6)	0.89	-
Diabetes mellitus	96 (17.4)	26 (18.6)	70 (17.0)	0.68	-
Hyperlipidaemia	169 (30.7)	31 (22.1)	138 (33.6)	0.01	0.01
BMI (kg/m^2^)	26.4 ± 5.6	25.6 ± 5.0	26.7 ± 5.8	0.07	-
Smoking history	155 (28.1)	44 (31.4)	111 (27.0)	0.32	-
Known cardiac disease	120 (21.8)	53 (37.9)	67 (16.3)	**0.008**	0.03
Prior stroke/ TIA	42 (7.6)	21 (15.0)	21 (5.1)	**0.0001**	0.03
**Blood fat profile**					
Cholesterol (mg/dL)	179.9 ± 32.7	156.5 ± 59.4	188.1 ± 53.4	**<0.0001**	0.06
LDL cholesterol (mg/dL)	110.0 ± 20.0	106.4 ± 43.1	111.4 ± 37.8	0.44	-
HDL cholesterol (mg/dL)	50.2 ± 9.1	48.9 ± 22.8	50.8 ± 16.5	0.54	-
Total cholesterol <152 mg/dL	169 (30.7)	69 (49.3)	100 (24.3)	**<0.0001**	0.06
Low cholesterol due to statin therapy	84 (15.2)	12 (8.6)	72 (17.5)	**0.01**	0.01
**Concomitant medication**					
Betablocker	206 (37.4)	48 (34.3)	158 (38.4)	0.34	-
ACE inhibitor/ARB	226 (41.0)	52 (37.1)	174 (42.3)	0.25	-
Anticoagulant	172 (31.2)	55 (39.3)	117 (28.5)	**0.02**	0.01
Platelet inhibition	118 (21.4)	32 (22.9)	86 (20.9)	0.66	-
Vit. D	109 (19.8)	30 (21.4)	79 (19.2)	0.59	-
Diabetes medication	77 (14.0)	21 (15.0)	56 (13.6)	0.66	-
Statin	136 (24.7)	29 (20.7)	107 (26.0)	0.19	-
**Cancer treatment**					
Platin	105 (19.1)	29 (20.7)	76 (18.5)	0.56	-
Taxane	157 (28.5)	39 (27.9)	118 (28.7)	0.85	-
Anthrazycline	147 (26.7)	44 (31.4)	103 (25.1)	0.14	-
Gemcitabine	24 (4.4)	11 (7.9)	13 (3.2)	**0.02**	0.01
5-FU	46 (8.3)	21 (15.0)	25 (6.1)	**0.001**	0.02
Aromatase inhibitors	45 (8.2)	11 (7.9)	34 (8.3)	0.88	-
Bortezomib/Lenalidomid	15 (2.7)	1 (0.7)	14 (3.4)	0.09	-
Surgical treatment	304 (55.2)	66 (47.1)	238 (57.9)	**0.03**	0.009
Radiation	213 (38.7)	69 (49.3)	144 (35.0)	**0.003**	0.02
Stem cell transplantation	26 (4.7)	12 (8.6)	14 (3.4)	**0.01**	0.01

Data are presented as mean ± SD or numbers (frequency). Median follow-up time is given as median (interquartile range). Univariable analysis was performed with linear regression analysis whereby death during follow-up served as dependent variable. Spearman’s correlation coefficient R^2^ was calculated as a coefficient of determination of the correlation. Bold values mark statistical significance. 5-FU: 5-fluorouracil; ACE: angiotensin converting enzyme; ARB: angiotensin receptor blocker; BMI: body mass index; CI: confidence interval; HDL: high density lipoprotein; LDL: low density lipoprotein; TIA: transitory ischemic attack; Vit.: Vitamin.

**Table 2:** Cholesterol levels based on different tumour entities. Values are presented as median [IQR].

	Entire Study Population	Patients Who Died	Alive during Follow-Up	*p*-Value
HPB	121 [99; 154]	143 [96; 160]	109 [98; 158]	0.84
Others	148 [136; 200]	108 [67; 148]	148 [147; 227]	0.13
ORL	151 [124; 205]	130 [96; 199]	152 [139; 211]	0.41
Kidney/Urothelium	157 [138; 199]	141 [121; 213]	160 [147; 199]	0.36
Sarcoma	159 [119; 220]	111 [109; 214]	193 [153; 228]	0.14
Lung	159 [117; 209]	171 [113; 219]	154 [118; 209]	0.91
Gastrointestinal	166 [122; 205]	135 [101; 227]	166 [127; 203]	0.9
Gynaecological	170 [137; 225]	158 [136; 173]	199 [134; 242]	0.17
Haematological	171 [113; 217]	115 [74; 177]	188 [137; 223]	**0.005**
Dermatological	174 [134; 206]	174	172 [133; 206]	0.97
Lymphoma	174 [147; 214]	139 [111; 161]	186 [165; 228]	0.02
Prostate	178 [133; 217]	160 [113; 196]	185 [148; 231]	0.18
Melanoma	194 [147; 247]	176 [124; 226]	194 [164; 256]	0.39
Cerebral	205 [164; 231]	128 [106; 150]	221 [204; 233]	**0.002**
Mamma	212 [163; 245]	160 [124; 228]	216 [180; 248]	**0.0004**

Cholesterol levels were compared between patients who died and who were alive during follow-up, across different tumour entities. Comparisons were performed using a two-tailed Student’s t-test. Bold values mark statistical significance. HPB: hepatopancreaticobiliary; IQR: interquartile range; ORL: otorinolaryngological.

**Table 3:** Cox regression analysis.

	HR	95% CI	*p*-Value
Age at first diagnosis	1.02	1.01 to 1.03	0.007
Metastasised tumour stage	3.31	2.24 to 4.88	<0.0001
Known cardiac disease	1.58	1.07 to 2.3	0.02
Prior stroke/TIA	1.73	1.06 to 2.82	0.03
Total cholesterol <152 mg/dL	1.70	1.21 to 2.39	0.002
Treatment with 5-FU	1.32	0.81 to 2.15	0.26
Radiation	0.95	0.66 to 1.37	0.78
Stem cell transplantation	4.31	2.27 to 8.20	<0.0001

5-FU: 5-fluorouracil; CI: confidence interval; CV: cardiovascular; HR: hazard ratio; TIA transitory ischemic attack.

## Data Availability

Data will be made available from the corresponding author upon reasonable request.
